# Unmasking Incontinentia Pigmenti: A Multimodal Clinico-Dermoscopic and Pathologic Correlation

**DOI:** 10.3390/diagnostics16162544

**Published:** 2026-08-12

**Authors:** Michał Niedźwiedź, Małgorzata Skibińska, Marcin Kurowski, Katarzyna Poznańska-Kurowska

**Affiliations:** 1 Department of Dermatology, Pediatric Dermatology and Oncology, Wł. Biegański Specialist Hospital, Kniaziewicza 1/5, 91-347 Lodz, Poland; malgorzata.skibinska@umed.lodz.pl (M.S.); katarzyna.poznanska@imp.lodz.pl (K.P.-K.); 2 Department of Immunology and Allergy, Medical University of Lodz, 90-419 Lodz, Poland; marcin.kurowski@umed.lodz.pl; 3 Nofer Institute of Occupational Medicine, sw. Teresy 8, 91-348 Lodz, Poland

**Keywords:** incontinentia pigmenti, dermoscopy, infant, newborn, skin diseases, vesiculobullous, genetic diseases, X-linked, retinal diseases, biopsy, genetic testing

## Abstract

Incontinentia pigmenti (IP) is a rare, X-linked dominant genodermatosis caused by mutations in the *IKBKG* gene and characterized by sequential cutaneous stages following the lines of Blaschko. The highly inflammatory initial vesiculobullous stage frequently mimics severe neonatal infections, such as herpes simplex or bullous impetigo, leading to potentially dangerous diagnostic delays and unnecessary antimicrobial therapies. The objective of this report is to highlight the utility of multimodal medical imaging and clinico-pathologic correlation in the rapid diagnosis of early-stage IP. We present the case of a full-term female neonate presenting on the third day of life with a rapidly disseminating blistering eruption, initially treated as a widespread infectious process. A multimodal diagnostic approach was applied, incorporating bedside clinico-dermoscopic evaluation of the infant and her mother (who reported a history of early miscarriages), followed by neonatal skin biopsy, immunohistochemistry (S-100, MART-1), and subsequent genetic testing. Dermoscopy of the neonate’s transitional lesions revealed early dermal melanophage accumulation, while maternal evaluation exposed pathognomonic residual Blaschko-linear dyspigmentation featuring a distinct “pepper-like” dermoscopic pattern. Histopathology demonstrated classic eosinophilic spongiosis, intraepidermal vesicles, and early pigment incontinence. Genetic testing confirmed recurrent *IKBKG* exon 4–10 deletion. Crucially, this rapid diagnosis facilitated immediate targeted ophthalmologic screening, detecting asymptomatic stage 2B retinal vasculopathy. Integrating noninvasive dermoscopy with precise histopathologic correlation offers a rapid, highly effective framework to distinguish early IP from neonatal blistering infections. Timely multimodal imaging is crucial for triggering immediate multidisciplinary screening, effectively preventing severe, irreversible vision-threatening complications.

**Figure 1:**
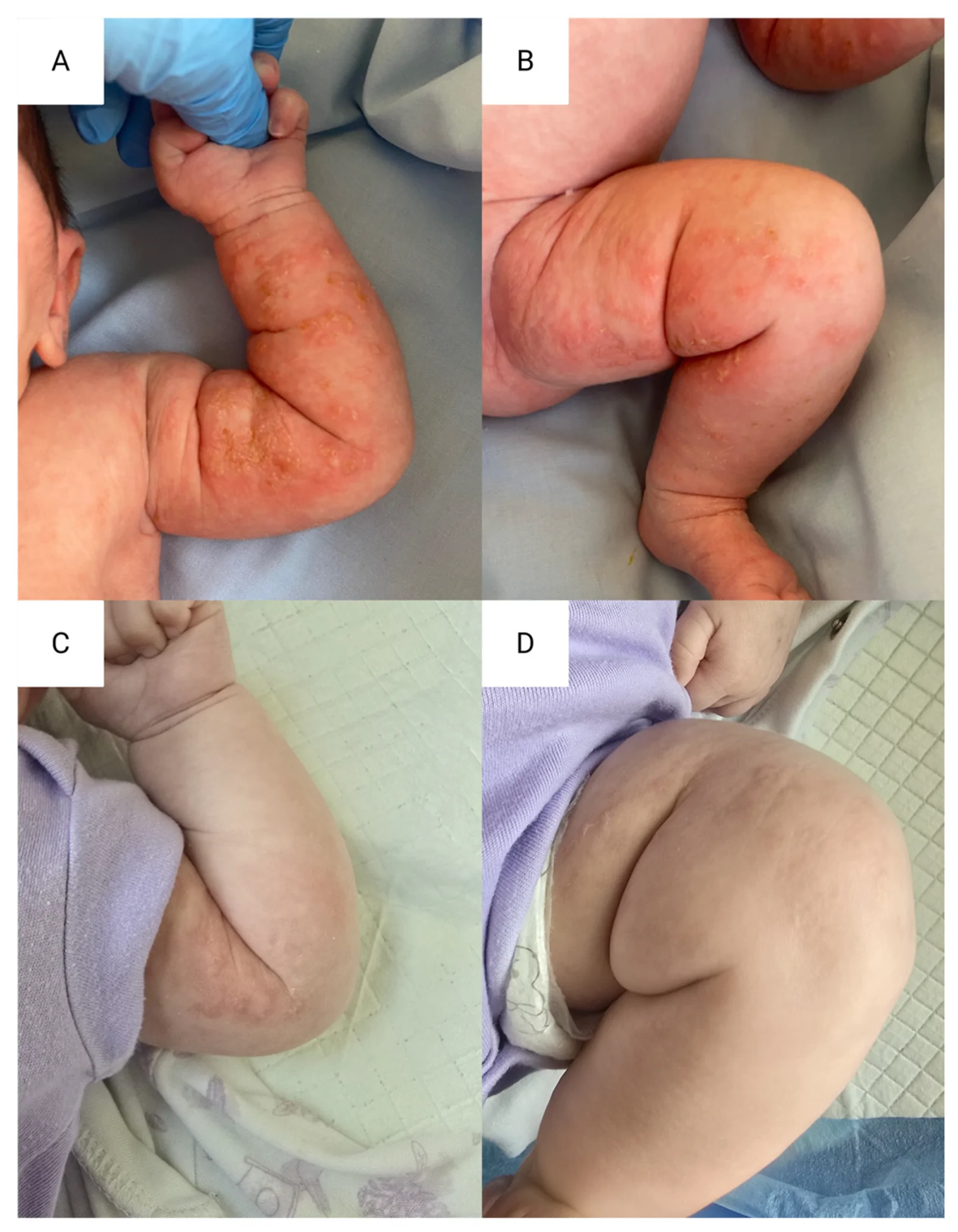
Incontinentia pigmenti (IP, Bloch–Sulzberger syndrome) is a rare X-linked dominant genodermatosis (estimated incidence ~0.7 per 100,000 live births), occurring almost exclusively in females [1,2,3]. It is usually lethal in utero for males carrying hemizygous complete loss-of-function variants. The disease is caused by heterozygous mutations in the *IKBKG* (formerly *NEMO*) gene at Xq28, which encodes a crucial regulatory subunit of the NF-κB signaling pathway essential for inflammation, immune function, and apoptosis [4]. IP is clinically characterized by an evolutionary sequence of skin lesions along Blaschko lines, resulting from functional somatic mosaicism due to random X-chromosome inactivation [2]. Cutaneous manifestations progress through four overlapping stages: vesicular (stage I), verrucous (stage II), hyperpigmented (stage III), and hypopigmented/atrophic (stage IV). While skin lesions often fade by adulthood, significant morbidity arises from extracutaneous manifestations affecting the eyes, central nervous system (CNS), teeth, and hair [1,2]. Early diagnosis is vital for initiating prompt surveillance, yet the neonatal vesicular stage is frequently misdiagnosed as an infectious or immune-bullous process [1,2]. Here, we present cases of early- and late-stage IP, highlighting the diagnostic journey, dermoscopic findings, and clinico-pathologic correlation supported by genetic testing. A full-term female neonate (39 weeks of gestation, birth weight 3860 g) developed an erythematous vesiculobullous eruption on the third day of life, initially on the right lower leg and dorsal foot. Within 48 h, lesions rapidly disseminated along Blaschko lines across all extremities and the trunk, evolving into superficial erosions with honey-colored crusts (**A**,**B**). Initially, due to suspected widespread infectious process (e.g., bullous impetigo or neonatal herpes simplex), the patient was admitted to the NICU. Empirical treatment with intravenous cefuroxime (50 mg/kg/d), topical 1% hydrocortisone, and 2% fusidic acid ointment failed to improve the skin lesions significantly. At 10 weeks of age, she was referred to the pediatric dermatology department. Examination revealed persistent vesiculobullous and emerging verrucous changes, predominantly on the proximal left arm, right leg, and left thigh (**C**,**D**), alongside early hyperpigmented streaks along Blaschko lines.

**Figure 2:**
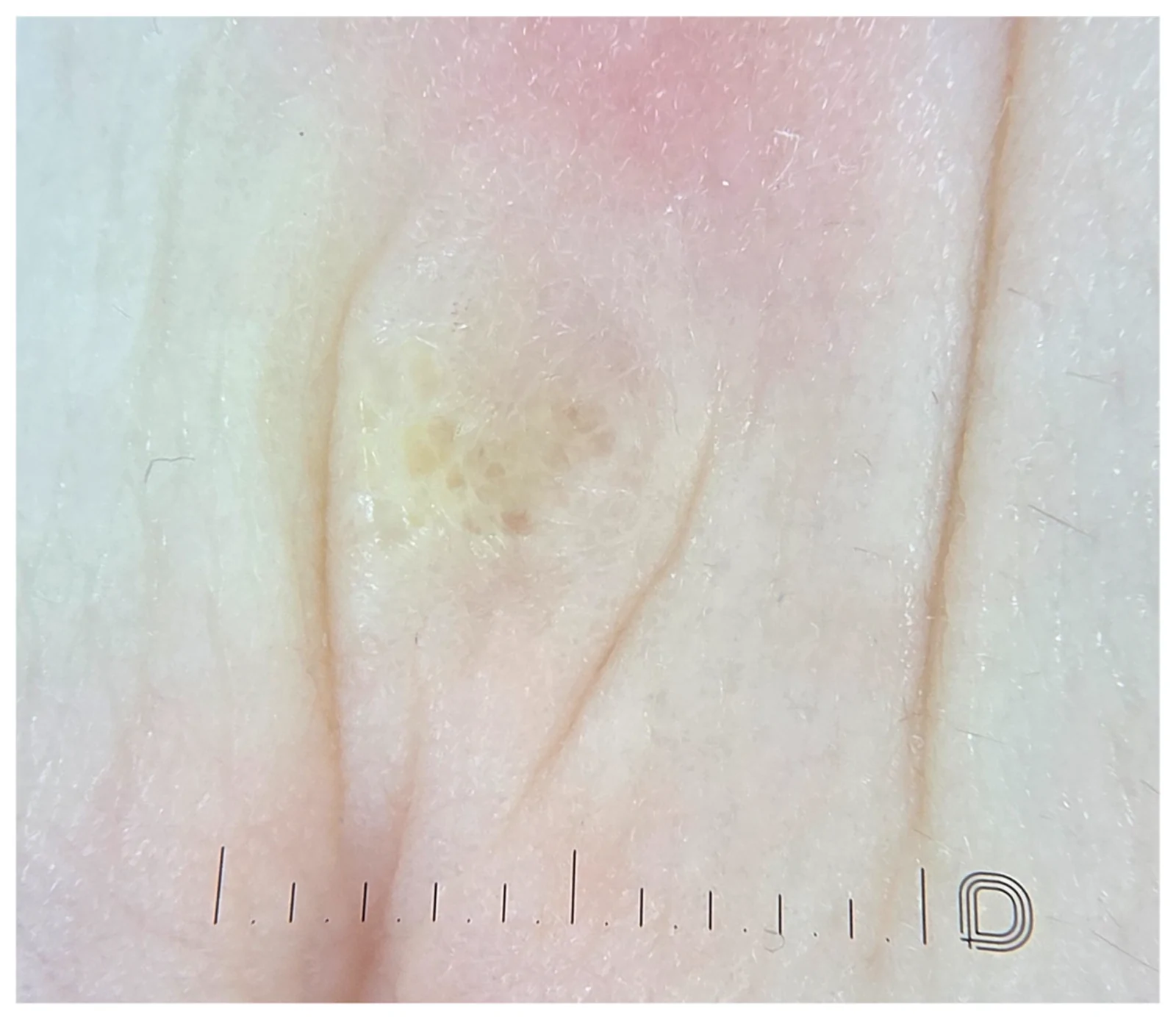
Dermoscopy of a transitional lesion on the medial left elbow (original magnification ×10, DermLite, DL5), performed prior to the skin biopsy. The image revealed a central yellowish structureless area—indicative of early hyperkeratosis and resolving exudate (Stage II)—containing a focal cluster of grey-brown dots and globules. These pigmented structures correspond to early dermal melanophage accumulation (transition to Stage III), all set against a faint erythematous background. This immediate bedside recognition of IP prompted targeted neurological and ophthalmologic screenings before histopathological and genetic confirmation.

**Figure 3:**
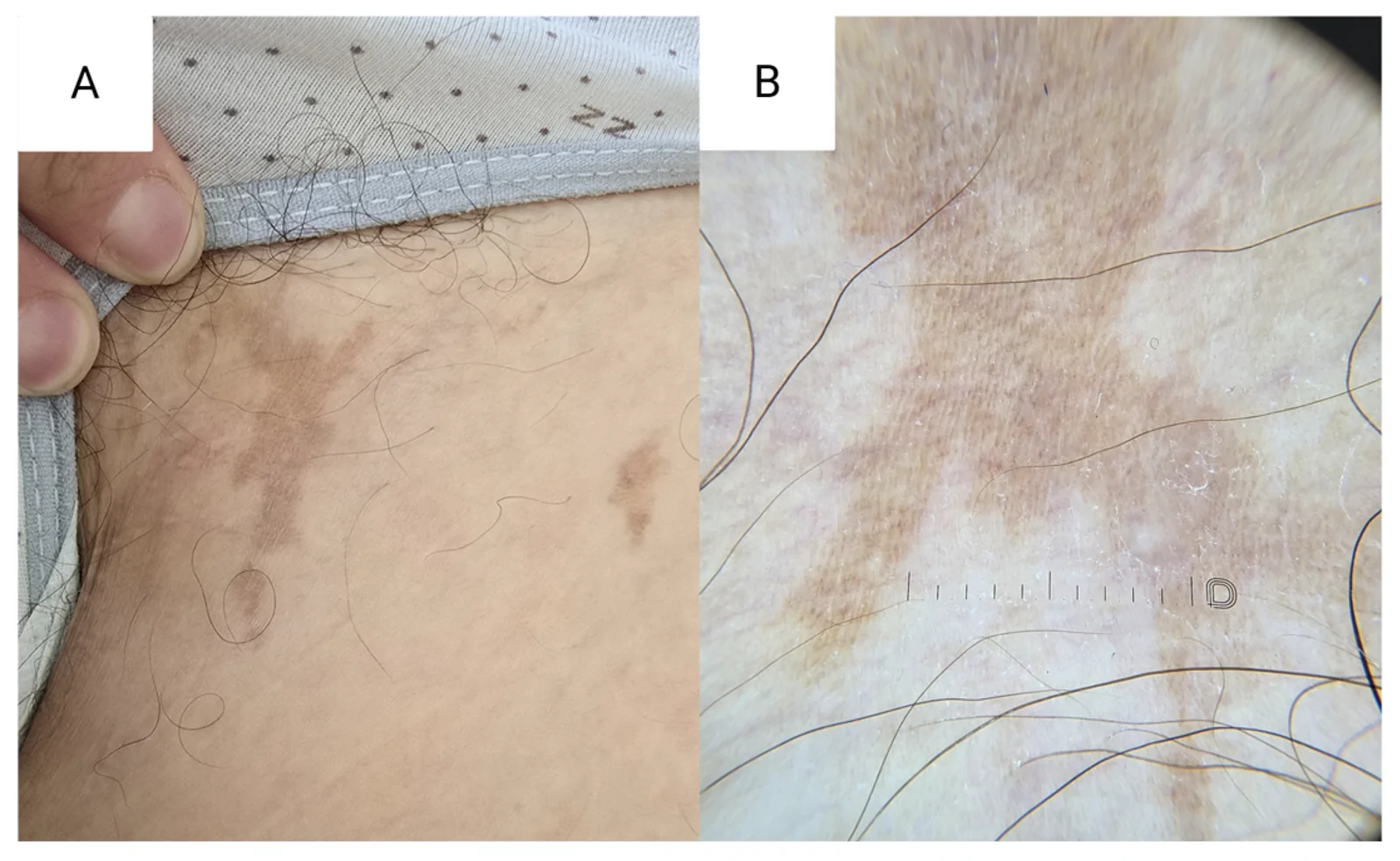
The 38-year-old mother reported a history of similar neonatal blistering eruption, initially misdiagnosed as Stevens–Johnson syndrome, which spontaneously resolved. The mother exhibited residual, linear hyperpigmented streaks along the lines of Blaschko on the right groin (**A**). Dermoscopy of the hyperpigmented area (original magnification ×10, DermLite DL5) revealed an arrangement of brown-to-grey dots and globules forming linear and vaguely reticular patterns on a lighter background (**B**). This specific distribution of dermal pigmentation is a classic hallmark of late-stage (Stage III) incontinentia pigmenti. Dermoscopy is increasingly recognized as a noninvasive additional tool for evaluating IP [5,6]. Early inflammatory lesions (Figure 2) typically display a polymorphous vascular pattern on an erythematous background with grey-brown dots (dermal melanophages) [5,6,7]. Stage III lesions (**B**) reveal a dense, “pepper-like” reticular network of brown and slate-grey dots, while atrophic stage IV areas show a homogeneous whitish background with adnexal loss [5,6]. Our observations align with these patterns, suggesting dermoscopy can unmask subtle Blaschko-linear distributions and document disease evolution in both probands (Figure 2) and relatives (**B**). In clinical practice, this noninvasive approach not only facilitates diagnosis but also provides valuable insights into monitoring disease progression. While the vesicular stage (Stage I) often presents with nonspecific vascular patterns, the early appearance of grey-brown structures, corresponding to dermal melanophages, may serve as a precursor to hyperpigmentation, appearing even before clinical manifestation [5,6,7]. Crucially, dermoscopy allows for the differentiation of IP from common infectious blistering disorders: unlike impetigo, which typically shows ‘honey-crust’ structures without disseminated melanophages, IP exhibits characteristic, albeit subtle, pigmentary patterns along the lines of Blaschko [5,7]. This distinction makes dermoscopy an invaluable tool supporting the differentiation from bacterial infections in neonates and preventing unnecessary antimicrobial therapies [7]. However, it must be emphasized that dermoscopy only assists in narrowing the differential diagnosis, and suspected infectious causes still strictly require appropriate clinical, laboratory, and microbiological evaluation.

**Figure 4:**
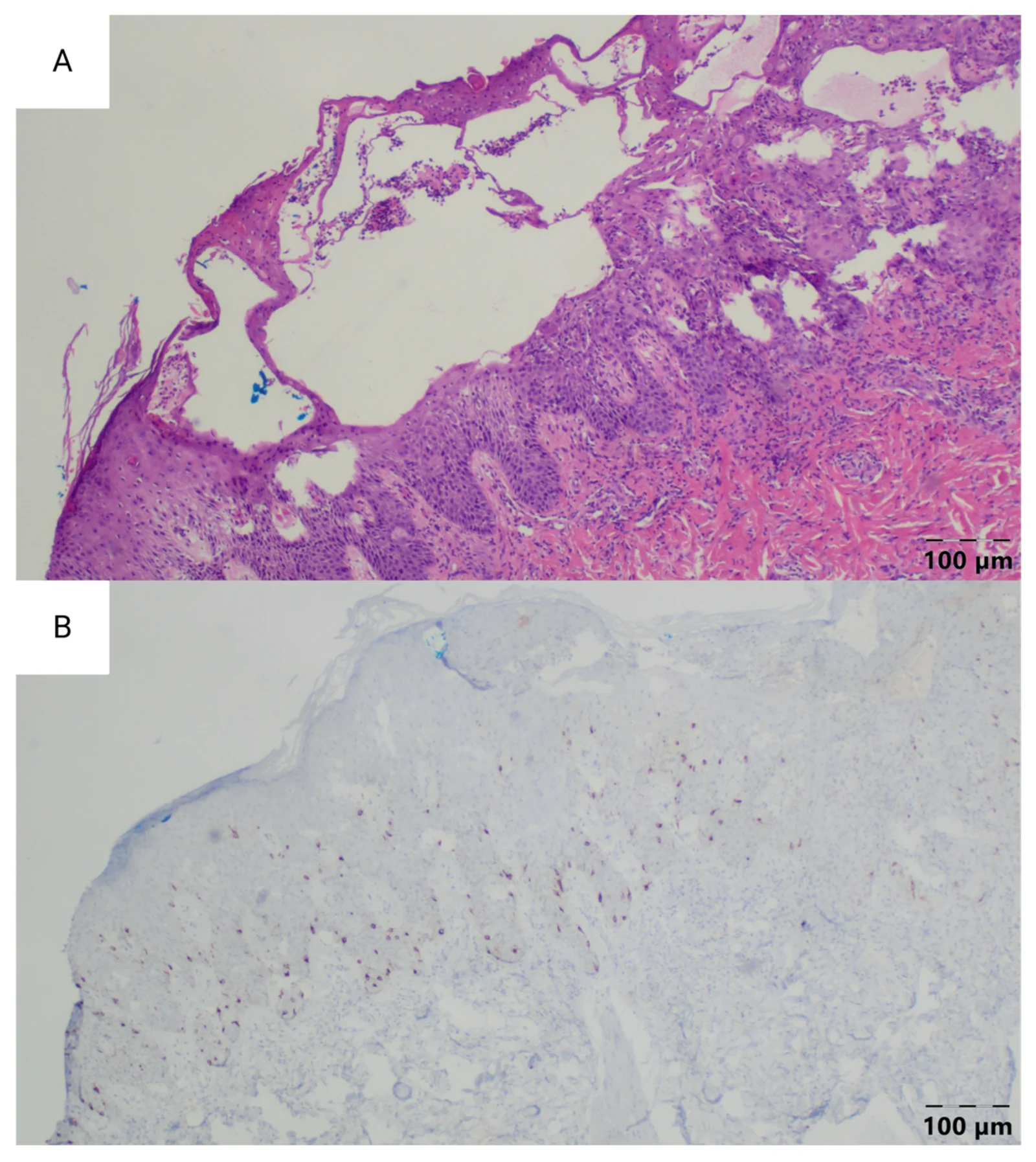
Skin biopsy specimen from the neonatal vesicular lesion demonstrated marked epidermal acanthosis, a large multilocular intraepidermal vesicle, prominent spongiosis, intravesicular and dermal eosinophils (hematoxylin–eosin; scale bar = 100 μm) (**A**). Immunohistochemical staining highlighted altered melanocyte distribution and early pigment incontinence, with positively stained cells scattered along the basal layer and descending into the superficial dermis (MART-1; scale bar = 100 μm) (**B**). Genetic testing using Multiplex Ligation-dependent Probe Amplification (MLPA) confirmed a recurrent pathogenic deletion of exons 4–10 in the *IKBKG* (NEMO) gene in both the patient and her mother, definitively establishing the diagnosis. A multidisciplinary screening protocol was initiated. While neurological and orthopedic evaluations were unremarkable, a dilated fundus examination revealed left retinal vascular anomalies (dilated peripheral veins with arteriovenous anastomoses), indicating stage 2B vasculopathy. The patient was discharged on topical emollients with strict follow-up. By 4 months of age, vesicular lesions had regressed into verrucous plaques and prominent hyperpigmentation along Blaschko lines. IP represents a significant diagnostic challenge in neonates, as stage I can closely mimic severe infectious and genetic blistering disorders [1]. The Blaschko-linear distribution (Figure 1A,B) marked tissue eosinophilia, and rapid evolution into verrucous and hyperpigmented stages distinguish IP from neonatal herpes simplex, bullous impetigo, staphylococcal scalded skin syndrome, or epidermolysis bullosa [2]. In our patient, this classic pattern, combined with eosinophilic spongiosis, dyskeratotic keratinocytes, and a suggestive X-linked family history, strongly supported the diagnosis before molecular confirmation. Identifying the recurrent *IKBKG* exon 4–10 deletion aligns with current genetic data, as this is the most frequent pathogenic variant, reported in 70–80% of affected individuals [4]. The marked phenotypic variability between our neonate and her mildly affected mother likely reflects skewed X-chromosome inactivation and the pleiotropic role of NF-κB signaling in ectodermal and neurovascular development [4]. The systemic nature of IP dictates prognosis and follow-up [2]. Cohort studies indicate CNS abnormalities in 15–35% of patients, ocular involvement in 35–70%, and dental/oral anomalies in 30–90% [2]. Neurologic complications, including neonatal seizures and ischemic strokes, account for substantial morbidity [8]. Ophthalmologic manifestations range from pigmentary retinal changes to peripheral nonperfusion, neovascularization, and tractional retinal detachment, with the risk of irreversible vision loss [8,9]. Furthermore, approximately one-third of female IP patients exhibit immunological manifestations, specifically the production of autoantibodies neutralizing type I interferons secondary to thymic dysplasia, which confers an increased susceptibility to life-threatening viral diseases [10]. Recent European consensus recommendations emphasize that timely detection and treatment of retinal vasculopathy can significantly reduce the risk of visual impairment [2]. Guidelines advocate urgent, repeated dilated indirect ophthalmoscopy in suspected IP, utilizing prompt peripheral laser photocoagulation when indicated. Parallel structured neurologic surveillance facilitates early intervention for children at risk of neurocognitive sequelae. Our patient’s early ophthalmologic findings emphasize that ocular involvement may already exist in otherwise asymptomatic neonates, necessitating immediate multidisciplinary care. Dermatologic management is largely supportive [2]. Most vesiculobullous and verrucous eruptions resolve spontaneously; treatment focuses on gentle skin care, emollients, and topical corticosteroids for severe inflammation [1,2]. Recognizing residual stage III and IV cutaneous changes in older relatives, often subtle or asymptomatic, serves as a critical diagnostic clue for evaluating at-risk family members and guiding genetic counseling [1,2]. The presented case demonstrates the practical utility of integrating dermoscopy with clinical and histopathological findings to facilitate early recognition of IP. By presenting a bedside-to-microscope correlation of the transitional phase in a neonate alongside the subtle residual findings in the mother, these images serve to reinforce the clinical recognition of this complex syndrome across different stages. As these findings are based on a single clinical observation, the broad diagnostic capacity of dermoscopy in early-stage IP requires validation in larger patient cohorts. In conclusion, IP must be considered in female neonates presenting with Blaschko-linear vesiculobullous eruptions, particularly alongside peripheral eosinophilia and a negative infectious workup [1,2,11]. Consistent with the broader literature, dermoscopy and histopathology provide rapid correlation with evolving clinicopathologic stages, enabling distinction of IP from other blistering disorders [5,6,7]. Early genetic confirmation triggers essential ophthalmologic, neurologic, and dental care pathways [5,6,7]. Current guidelines emphasize that a high index of suspicion and multidisciplinary collaboration are imperative to prevent avoidable visual and neurologic disability in this neurocutaneous syndrome [2].

## Data Availability

No new data were created or analyzed in this study. Data sharing is not applicable to this article.

## Abbreviations

The following abbreviations are used in this manuscript:

CNS	Central Nervous System
IP	Incontinentia Pigmenti
*IKBKG*	Inhibitor of Nuclear Factor Kappa-B Kinase Subunit Gamma
S-100	S100 Proteins
MART-1	Melanoma Antigen Recognized by T cells 1
MLPA	Multiplex Ligation-Dependent Probe Amplification
NEMO	NF-Kappa-B Essential Modulator
NF-κB	Nuclear Factor Kappa-Light-Chain-Enhancer of Activated B Cells
NICU	Neonatal Intensive Care Unit
